# Changing school start times: impact on sleep in primary and secondary school students

**DOI:** 10.1093/sleep/zsab048

**Published:** 2021-04-15

**Authors:** Lisa J Meltzer, Kyla L Wahlstrom, Amy E Plog, Matthew J Strand

**Affiliations:** 1 National Jewish Health, Denver, CO, USA; 2 University of Minnesota, Minneapolis, MN, USA; 3 Cherry Creek School District, Greenwood Village, CO,USA

**Keywords:** school start times, sleep, health policy, longitudinal, adolescent, elementary

## Abstract

**Study Objectives:**

To examine the impact of changing school start times on sleep for primary (elementary school: ES) and secondary (middle and high school: MS/HS) students.

**Methods:**

Students (grades 3–12) and parents (grades K-12) were surveyed annually, before and for 2 years after school start time changes (ES: 60 min earlier, MS: 40–60 min later; HS: 70 min later). Student sleep and daytime sleepiness were measured with school-administered student surveys and parent-proxy online surveys.

**Results:**

Approximately 28,000 students annually completed surveys (~55% White, ~21% free/reduced lunch [FRL]). One-year post-change, weekday bedtimes and wake times were slightly earlier for ES students, with an 11-min decrease in sleep duration. MS and HS students reported slightly later weekday bedtimes, significantly later wake times, and significantly longer sleep duration (MS: 29 min; HS: 45 min). The percent of ES students reporting sufficient sleep duration, poor sleep quality, or daytime sleepiness did not change, but the percent of MS and HS students reporting sufficient sleep duration significantly increased and clinically significant daytime sleepiness decreased. All results were maintained at the 2-year follow-up. Benefits of later start times were similar across racial and free/reduced lunch groups.

**Conclusions:**

This is the first large scale, longitudinal, and representative study to concurrently examine the impact of changing school start times across students in primary/secondary school. Findings suggest a minimal impact of earlier start times on ES students’ sleep or daytime sleepiness, while further supporting the significant benefits of delaying MS and HS start times on student sleep and daytime sleepiness.

**Statement of Significance**

This study highlights the significant benefit of later school start times for middle school and high school students, while also demonstrating no significant negative effects of earlier elementary school start times. The study is novel due to the large sample size, the 2-year follow-up period, and the relatively diverse sample. The implementation of healthy school start times (at or after 8:30 am for middle and high school students) is a critical health policy that can quickly and effectively address significant adolescent sleep debt, with minimal impact on younger students, who often are required to start earlier in order to accommodate later secondary school start times. Future studies should examine how early is too early to start school across development.

## Introduction

Sleep is essential for optimal health and development, academic achievement, and social and emotional functioning [1–3], yet insufficient sleep is common among children and adolescents. To address this public health concern, the goal of increasing sleep duration for not only adolescents, but for children of all ages, was recently identified as a target for Healthy People 2030 [4]. For secondary school students, healthy school start times (no earlier than 8:30 am) have been identified by most major professional associations, including the American Academy of Pediatrics [5], American Academy of Sleep Medicine [6], American Medical Association [7], American Psychological Association [8], National Association of School Nurses [9], and National Parent Teacher Association [10], as a modifiable policy to improve adolescent health and well-being. The need for, and benefits of, later secondary school start times is well documented [1, 11, 12]. However, it is notable that <21% of middle schools and <18% of US high schools start at 8:30 am or later [13, 14]. Thus, further evidence supporting later start times for adolescents is needed.

In order to achieve later secondary school start times, it is often necessary for primary school students to start earlier, especially if the district has a staggered transportation schedule and has only one set of buses that transports all students [15]. Based on biological changes to human circadian rhythms during puberty, early sleep onset is difficult for adolescents, whose sleep is then truncated with early school start times, resulting in deficient sleep [16]. Primary school students, on the other hand, typically go to bed and wake earlier than secondary school students [17, 18]. Furthermore, biological changes in the circadian rhythm and self-reported delays in circadian preference (i.e. time one feels most alert and awake) typically begin between 11 and 13 years, when students in the United States are transitioning from elementary to middle school [19, 20]. Studies have shown an increase in sleep duration for primary school-age students based on changing to an earlier bed time and maintaining a consistent wake time [21–23]. Together, these studies suggest primary school students are able to fall asleep earlier if they have an earlier bedtime set for them, allowing them the opportunity to obtain sufficient sleep duration, even with an earlier school start time. However, bedtimes are often set by parents, and are part of greater family routines and schedules. Thus, even though primary school students are biologically able to fall asleep earlier than adolescents, few studies have considered whether the implementation of earlier primary school start times (as a result of delayed secondary school start times) results in changes to sleep routines and sleep duration.

Only two studies in the United States have prospectively examined how district changes to start times impact primary school students’ sleep [24, 25]. Qualitative findings from a large, diverse urban district in Minnesota reported that moving to later start times (8:40–9:40 am) negatively impacted transportation, student behavior, staff meetings, morning teaching and learning, and afternoon student fatigue. Schools that moved earlier (9:40 am to either 8:40 am or 7:40 am) reported students benefitting from fewer transitions before school, having fewer behavior problems, and being more alert and focused during the day [24]. In the northeast United States, minimal changes in sleep were found after moving start times earlier (3rd grade: 9:10–7:45 am, 4th and 5th grade: 8:20–7:45 am). Students reported earlier bedtimes and wake times, increased sleep duration for 3rd graders (24 min), minimally decreased sleep duration for 4th and 5th graders (4 and 9 min, respectively), and no change in daytime sleepiness [25]. However, the study sample included only one school that was predominantly white (97.8%) and not economically diverse. No studies have concurrently considered the impact on changing start times on sleep for students across grades from Kindergarten through 12th grade.

Finally, questions also remain about whether the impact of changing school start times is consistent across different racial and economic groups of students. Many studies that have looked at the impact of changes to school start times on sleep have focused on schools where the majority of students were non-Hispanic white and/or did not qualify for free or reduced lunch (FRL), a proxy schools often use for poverty (e.g. Refs. [25–28]). Across studies, even those with somewhat more diverse samples, the impact of changing start times on sleep by race or FRL status has not been considered. However, both racial minority and low socioeconomic status students at all levels may be disproportionately impacted by early start times (e.g. unable to get to school if they oversleep and miss the bus) [29], thus, it is important to consider these variables.

As a result, there remains a significant need for rigorous, longitudinal research that includes large, diverse samples, to demonstrate how this important health policy impacts *all* students from Kindergarten through 12th grade. In August 2017, the Cherry Creek School District (CCSD), a diverse district of ~55,000 students in suburban Denver, Colorado, changed school start times, delaying high schools (HS, grades 9–12, typical ages 14–18 years) by 70 min (to 8:20 am) and middle schools (MS, grades 6–8, typical ages 11–14 years) by 40–60 min (to 8:50 am), while moving elementary schools (ES, grades K-5, typical ages 5–11 years) 60 min earlier (to start at 8:00 am) [30]. To address knowledge gaps noted above, the Changing Start Times: Longitudinal Effects Study (CaSTLES) was developed to evaluate outcomes in the CCSD before and for 2 years after the start time change. The aim of this paper is to examine the impact of changing school start times on student sleep, including bedtime, wake time, sleep duration, sleep quality, and daytime sleepiness on students K-12.

## Methods

### Study design and participants

Students enrolled in the CCSD and their parents were invited to participate in CaSTLES in spring 2017 (pre-change, ~4 months before start times changed), spring 2018 (post-change, ~6 months post-change), and once again in spring 2019 (follow-up, ~18 months post-change). Pre-change surveys included students in grades 3 through 11 (and parents of students in Kindergarten [K] through 11th grade), and both the post-change and follow-up surveys included students in grades 3 through 12 (and parents of students in K through 12th grade). There were no exclusion criteria. Approximately 24,000–30,000 students chose to participate in the study each year. Study surveys and procedures were approved by the CCSD Research Review Committee, and all applicable ethical standards were followed. Parents were informed of the upcoming survey through multiple email and phone notifications from CCSD in their preferred language (i.e. English, Amharic, Arabic, Chinese, Korean, Russian, Spanish, and Vietnamese), with the option to have their student not participate; less than one-half of one percent of all parents in the district opted out of the study. Student surveys were administered on laptops or tablets during designated class periods. Prior to starting the survey, students were informed both verbally and within the survey that participation was voluntary. If needed, survey questions/responses were read aloud to students. Parent surveys were sent via email (in the parent’s preferred language), with multiple email and phone reminders to complete the survey. Some families had two parents or caregivers complete surveys, but only the primary caregiver report is included in this paper. Survey data were collected with SurveyGizmo (Boulder, CO). Demographic information was provided by the school district, including race and FRL status.

### Outcome measures

#### Sleep timing and duration

Student and parent surveys separately asked about students’ typical bedtime and wake time on both weekdays and weekends. Times were selected from drop down menus in 5 min increments, providing the following outcomes for weekdays and weekends: (1) bedtime; (2) wake time; (3) sleep duration (hours between bedtime and wake time); and (4) weekend oversleep (difference between weekday and weekend sleep duration).

#### Sleep quality and daytime sleepiness

Three items from the PROMIS Pediatric Sleep Disturbances item bank and two items from the PROMIS Pediatric Sleep Related Impairment item bank were included to measure sleep quality and daytime sleepiness [31]. All questions had a 7-day recall period (*In the past 7 days*…), and used a 5-point Likert response scale (“Always” to “Never”). *T*-scores (mean of 50 and standard deviation of 10) were derived based on national normative data through the Health Measures Scoring Service (assessmentcenter.net/ac_scoringservice).

### Data analysis

As this was an anonymous survey, it was not possible to link the full sample of students across years. Therefore, we utilized three approaches for examining the data. The *ecological approach* was our primary analysis, with data averaged within level, school, race, and year. Sleep outcomes were then fit as a function of race, year, and race-by-year, using linear mixed models, stratified by levels (ES, middle school [MS], high school [HS]). To account for correlated data within schools, a Kronecker Product structure was used for the error covariance structure, with an Unstructured covariance structure for race (5 levels: White, Black, Hispanic, Asian, Mixed Race/American Indian or Alaskan Native/Native Hawaiian or Other Pacific Islander [MR/AIAN/NHOPI]) and a first-order autoregressive (AR(1)) structure for year (pre, post, follow-up). This model allowed us to examine whether changes over time in mean sleep outcomes were modified by race. Since some schools had more participants than others, each outcome mean was weighted by the number of subjects used in the average. A second set of models, using a similar approach, were used to test for free and reduced lunch status (FRL, 2 levels: yes or no) as a predictor. Using the methods described above, we carried out tests to determine whether there were significant race-by-time or FRL-by-time interactions.

We also examined the data using a *unit-level approach*, with models fit as previously described, but using individual subjects in the model fit, and correlated data were accounted for by including a random intercept for school (rather than a non-simple error covariance structure). Results were similar to the *ecological approach*, and unless otherwise noted, are not reported. The third approach was *descriptive*, with means and asymptotic confidence intervals computed for each year (by level); however, this approach does not allow for statistical comparisons between years.

In addition, descriptive statistics were used to identify the proportion of students each year who (1) were obtaining sufficient sleep, (2) had poor sleep quality, or (3) had significant daytime sleepiness. Sufficient weekday sleep duration for ES and MS students was defined as at least 9 h, and for HS students as at least 8 h [4]. Poor sleep quality and significant daytime sleepiness were defined as a *T*-score ≥ 60 (1 SD above the mean) on the PROMIS measures [31, 32]. When reporting student-reported outcomes, parent-proxy data for younger ES students (grades K-2) are provided. Parent-proxy data for older ES students (grades 3–5), MS, and HS students are reported separately. Given the large study sample size and number of analyses, findings were considered significant if *p* < 0.001.

## Results

### Sample characteristics

Sample demographics are shown in Table 1. Older ES (grades 3–5) student participation was consistent across years (~77%), with increased participation over time in MS (~70%–79%) and HS students (52%–60%). Notably fewer parents of younger ES students (grades K-2) participated (~28%–37%). The sample was similar to the district in terms of gender, although some groups were less representative of students who qualify for FRL (district average 29.0%) or Hispanic students (district average 20.1%). However, this was not consistent across years or levels.

**Table 1.** Demographic characteristics of students

	Younger elementary school (Grades K-2)			Older elementary school (Grades 3–5)		
	Pre-change	Post-change	Follow-up	Pre-change	Post-change	Follow-up
Total *N*	4,207	3,159	3,149	9,604	9,812	9,720
% enrolled	37.2	27.9	27.5	77.0	77.3	77.8
% female	52.6	53.8	52.0	47.6	48.6	48.7
% FRL	18.4	16.4	15.6	22.3	27.4	18.7
Grade						
% K	31.3	30.4	31.1			
% 1st	34.1	35.3	34.8			
% 2nd	34.6	34.3	34.1			
% 3rd				34.0	31.7	30.3
% 4th				33.7	34.8	33.6
% 5th				32.4	33.5	36.1
Race/ethnicity						
% White	63.1	63.6	63.9	57.9	55.0	51.8
% Black	5.2	4.4	4.4	9.5	10.4	10.2
% Hispanic	14.4	14.4	14.4	16.1	17.5	20.4
% Asian	8.6	8.3	8.6	8.1	8.1	8.5
% MR/AIAN/NHOPI	8.8	9.3	8.7	8.4	9.0	9.1
	Middle school (Grades 6–8)			High school (Grades 9–12)		
	Pre-change	Post-change	Follow-up	Pre-change	Post-change	Follow-up
Total *N*	8,414	9,619	9,915	6,275	9,849	10,516
% Enrolled	69.6	79.1	75.7	51.9	59.8	60.4
% Female	51.0	49.8	49.1	52.6	51.2	51.3
% FRL	20.2	24.6	18.1	17.8	21.3	15.3
Grade						
% 6th	35.3	31.8	33.3			
% 7th	32.8	34.3	33.8			
% 8th	31.9	34.0	32.9			
% 9th				39.7	30.1	26.9
% 10th				33.0	28.6	26.2
% 11th				27.3	24.6	26.0
% 12th					16.7	20.9
Race/ethnicity						
% White	58.7	56.6	53.1	57.0	56.4	52.0
% Black	10.3	11.2	10.4	11.2	11.3	11.3
% Hispanic	15.1	16.7	20.5	15.4	16.9	19.9
% Asian	8.9	8.2	8.8	9.8	9.7	10.1
% MR/AIAN/NHOPI	6.9	7.2	7.3	6.5	6.6	6.8

FRL, free or reduced lunch status; MR/AIAN/NHOPI, mixed race/American Indian or Alaskan Native/Native Hawaiian or Other Pacific Islander.

### Sleep outcomes: full sample

**Figure 1.**
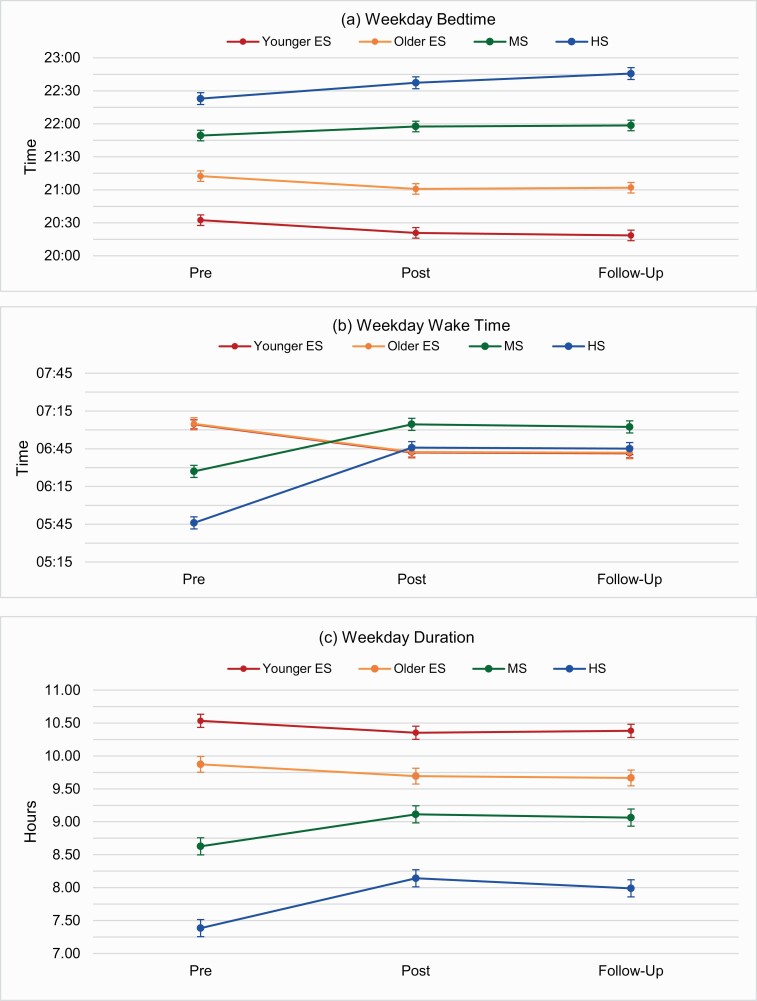
Changes in weekday sleep outcomes by school level using descriptive analyses.

Complete results using descriptive analyses for sleep outcomes by level are found in Table 2, with graphic representation of weekday bedtime, wake time, and sleep duration by level found in Figure 1. After the start time change (ES starting 60 min earlier), younger ES students had earlier average weekday bedtimes (11 min) and wake times (22 min), and decreased average weekday sleep duration (11 min). On weekends, younger ES students had average bedtimes and wake times that were earlier (11 and 10 min, respectively), with no difference in weekend duration. Average weekday and weekend sleep timing and duration remained relatively consistent at follow-up (change ≤4 min). Weekend oversleep increased by an average of 12 min at post-change. The percent of younger ES students obtaining sufficient sleep duration or having poor sleep quality did not differ at post-change or follow-up, with only a small increase in the percent of students reporting daytime sleepiness (4%) at post-change. However, at follow-up, the percent of students reporting daytime sleepiness was lower than pre-change.

**Table 2.** Means (95% CIs) and differences for sleep outcome variables across years using descriptive analytical methods

				*Difference*	
	Pre-change	Post-change	Follow-up	Post–Pre	Follow-up–Post
Younger elementary school (Grades K-2)					
Bedtime*					
Weekday	20:32 (20:31–20:34)	20:21 (20:19–20:22)	20:19 (20:17–20:20)	–11 min	–2 min
Weekend	21:10 (21:09–21:12)	20:59 (20:57–21:01)	20:56 (20:54–20:58)	–11 min	–3 min
Wake time*					
Weekday	7:04 (7:03– 7:06)	6:42 (6:41–6:43)	6:41 (6:40–6:43)	–22 min	–1 min
Weekend	7:42 (7:41–7:44)	7:32 (7:30–7:34)	7:28 (7:26–7:30)	–10 min	–4 min
Duration*					
Weekday	10.53 h (10.51–10.56)	10.35 h (10.33–10.38)	10.38 h (10.36–10.41)	–11 min	+2 min
Weekend	10.54 h (10.51–10.56)	10.55 h (10.52–10.58)	10.53 h (10.49–10.56)	+ <1 min	–2 min
Weekend oversleep^*,†^	0.2 min (–1.3 to 1.6)	12.1 min (10.3–13.9)	8.7 min (7.0–10.5)	+11.9 min	–3.4 min
% sufficient sleep^‡^	98.6%	98.2%	98.7%	–0.4%	+0.5%
% poor sleep quality^§^	22.3%	21.0%	19.7%	–1.3%	–1.3%
% daytime sleepiness^||^	34.8%	38.8%	33.0%	+4.0%	–5.8%
Older elementary school (Grades 3–5)					
Bedtime*					
Weekday	21:13 (21:11–21:14)	21:01 (21:00–21:02)	21:02 (21:01–21:03)	–12 min	+1 min
Weekend	22:21 (22:19–22:23)	22:19 (22:17–22:21)	22:20 (22:18–22:22)	–2 min	+1 min
Wake time*					
Weekday	7:05 (7:04–7:06)	6:42 (6:41–6:43)	6:42 (6:41–6:43)	–23 min	No change
Weekend	8:33 (8:31–8:35)	8:33 (8:31–8:35)	8:33 (8:31–8:35)	No change	No change
Duration*					
Weekday	9.87 h (9.85–9.90)	9.69 h (9.67–9.72)	9.67 h (9.64–9.69)	–11 min	–1 min
Weekend	10.19 h (10.16–10.23)	10.23 h (10.19–10.26)	10.22 h (10.18–10.25)	+2 min	– <1 min
Weekend Oversleep^*,†^	14 min (12–16)	26 min (24–28)	28 min (26–30)	+12 min	+2 min
% Sufficient sleep^‡^	83.5%	81.2%	80.2%	–2.3%	–1.0%
% Poor sleep quality^§^	24.1%	25.8%	23.8%	+1.7%	–2.0%
% Daytime sleepiness^||^	22.4%	26.5%	25.8%	+4.1%	–0.7%
Middle school (Grades 6–8)					
Bedtime*					
Weekday	21:49 (21:48–21:51)	21:58 (21:56–21:59)	21:59 (21:57–22:00)	+9 min	+1 min
Weekend	23:22 (23:20–23:24)	23:32 (23:30–23:34)	23:30 (23:28–23:32)	+10 min	–2 min
Wake time*					
Weekday	06:27 (06:26–06:28)	07:04 (07:04–07:05)	07:02 (07:02–07:03)	+37 min	–2 min
Weekend	09:12 (09:10–09:14)	09:20 (09:18–09:22)	09:16 (09:14–09:18)	+8 min	–4 min
Duration*					
Weekday	8.63 h (8.60–8.65)	9.11 h (9.09–9.14)	9.06 h (9.04–9.09)	+29 min	–3 min
Weekend	9.83 h (9.79–9.86)	9.79 h (9.76–9.82)	9.78 h (9.75–9.81)	–2 min	– <1 min
Weekend oversleep^*,†^	1.13 h (1.10–1.17)	0.60 h (0.57–0.63)	0.64 h (0.61–0.68)	–32 min	+2 min
% Sufficient sleep^‡^	40.5%	61.0%	59.6%	+20.5%	–1.4%
% Poor sleep quality^§^	30.3%	27.7%	28.9%	–2.6%	+1.2%
% Daytime sleepiness^||^	48.7%	37.1%	38.5%	–11.6%	+1.4%
High school (Grades 9–12)					
Bedtime*					
Weekday	22:23 (22:21–22:25)	22:37 (22:36–22:39)	22:46 (22:44–22:47)	+14 min	+9 min
Weekend	23:47 (23:45–23:50)	00:02 (00:01–00:04)	00:06 (00:05–00:08)	+15 min	+4 min
Wake time*					
Weekday	05:46 (05:45–05:47)	06:46 (06:45–06:47)	06:45 (06:44–06:46)	+60 min	–1 min
Weekend	09:16 (09:14–09:19)	09:27 (09:25–09:29)	09:27 (09:25–09:29)	+11 min	No change
Duration*					
Weekday	7.39 h (7.36–7.41)	8.14 h (8.12–8.17)	7.99 h (7.97–8.01)	+45 min	–9 min
Weekend	9.48 h (9.44–9.52)	9.41 h (9.38–9.44)	9.34 h (9.31–9.37)	–4 min	–4 min
Weekend oversleep^*,†^	2.05 h (2.01–2.09)	1.21 h (1.18–1.24)	1.28 h (1.25–1.31)	–77 min	+4 min
% Sufficient sleep^‡^	30.4%	62.7%	57.6%	+32.3%	–5.1%
% Poor sleep quality^§^	43.1%	31.2%	35.3%	–11.9%	+4.1%
% Daytime sleepiness^||^	76.2%	55.2%	62.0%	–21.0%	+6.8%

h = hours, min = minutes.

*Data are presented as mean (95% CI), with military time used for bedtimes and wake times.

^†^Weekend oversleep is the difference between weekday and weekend sleep duration.

^‡^Sufficient sleep defined as an average of at least 9 h for ES and MS, and at least 8 h for HS^4^.

^§^Poor sleep quality defined as *T* ≥ 60 on PROMIS Pediatric Sleep Disturbance items.

^||^Daytime sleepiness defined as *T* ≥ 60 on PROMIS Pediatric Sleep Related Impairment items.

For older ES students, a similar change was reported post-change for both average weekday bedtime (12 min earlier) and wake time (23 min earlier), resulting in a decrease in average weekday sleep duration of 11 min. There were no significant differences post-change in average weekend bedtimes, wake times, or sleep duration. Both average weekday and weekend sleep timing and duration remained consistent at follow-up. Average weekend oversleep increased by 12 min at post-change. The percent of older ES students reporting sufficient sleep duration slightly decreased (~2%) at post-change, while the percent of students reporting poor sleep quality or daytime sleepiness slightly increased (~2% and ~4%, respectively).

Post-change (MS starting 40–60 min later), MS students reported a slightly later average weekday bedtime (9 min), but a much later average weekday wake time (37 min), resulting in a clinically meaningful increase in average weekday sleep duration of 29 min. Average weekend bedtimes and wake times were slightly later post-change (10 and 8 min, respectively), resulting in no notable change in mean weekend sleep duration. Both average weekday and weekend timing and duration changes were maintained at follow-up. Weekend oversleep decreased post-change by an average of 32 min, with this difference maintained at follow-up. The percent of MS students obtaining sufficient sleep increased by ~21% post-change. Although there was only a small decrease in the percent of students with poor sleep quality (~3%), there was a notable decrease (~12%) in MS students reporting daytime sleepiness post-change. These differences were maintained at follow-up.

Finally, post-change (HS starting 70 min later) HS students reported a slightly later average weekday bedtime (14 min), but a later average weekday wake time (60 min), resulting in a clinically meaningful increase in average weekday sleep duration of 45 min. Average weekend bedtimes and wake times were also later (15 and 11 min, respectively), resulting in a relatively consistent average weekend sleep duration. Average weekday and weekend sleep timing and duration changes were maintained at follow-up. Average weekend oversleep was significantly reduced (77 min), with the percent of HS students with clinically significant oversleep (≥2 h) on weekends reduced from 54.6% pre-change to 31.4% at post-change and 33.2% at follow-up. The percent of HS students obtaining sufficient weekday sleep increased ~32% at post-change, with fewer students reporting poor sleep quality (~12%) and daytime sleepiness (21%) post-change.

For older ES, MS, and HS students, parent-proxy sleep outcomes are found in Supplemental Table 1 (ST-1). Overall at post-change, parent-reported sleep was similar to student-reported sleep, with a decrease in average weekday sleep duration for older ES (17 min) and a clinically significant increase in average weekday sleep duration for both MS (30 min) and HS (43 min), with no significant change in average weekend sleep duration for all three groups. Parents also reported a slightly earlier average weekday bedtime at post-change for ES students (9 min), with a slightly later mean weekday bedtime for MS students (6 min) and HS students (12 min). Average weekday wake times were earlier at post-change for older ES students (26 min), and later for MS students (36 min) and HS students (56 min). Finally, similar to student-report, parents reported an increase in average weekend oversleep for older ES students (18 min), but a decrease in average weekend oversleep for MS students (29 min) and HS students (46 min).

### Sleep outcomes by race and FRL status

**Figure 2.**
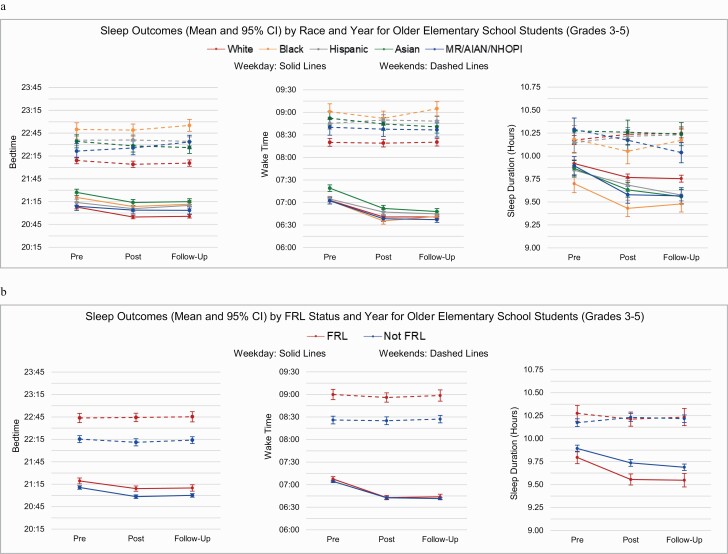
Sleep outcomes (mean and 95% CI) for later elementary school students (grades 3–5); (A) by race and year; (B) by FRL status and year using ecological analyses.

**Figure 3.**
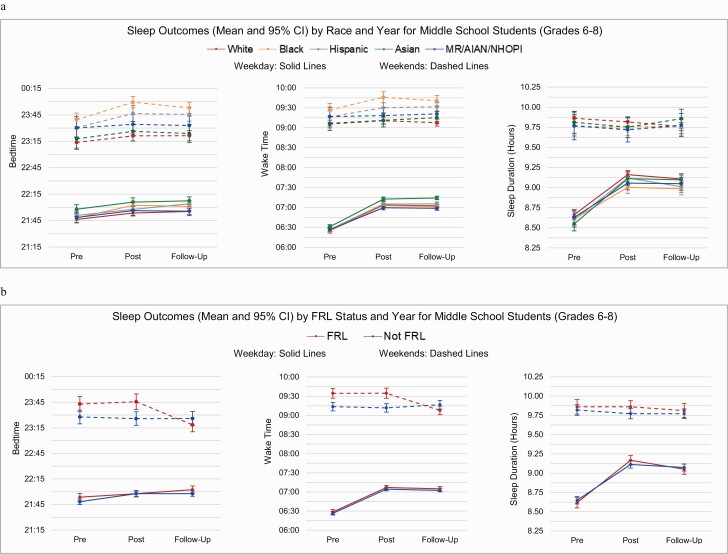
Sleep outcomes (mean and 95% CI) for middle school students (grades 6–8); (A) by race and year; (B) by FRL status and year.

**Figure 4.**
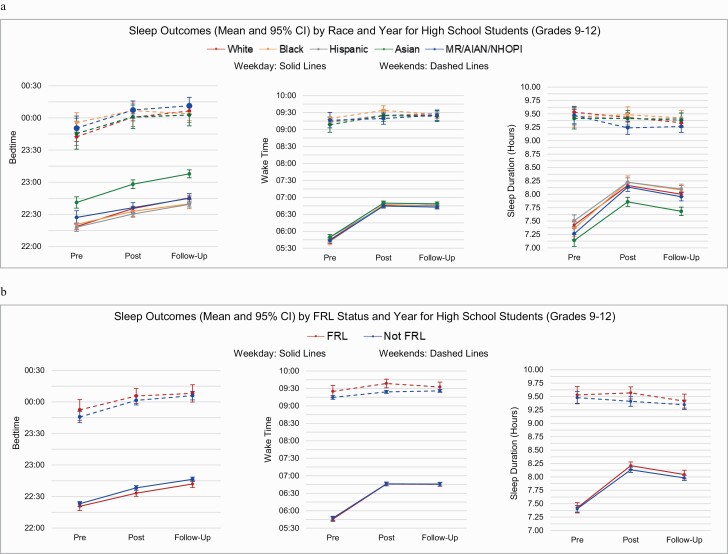
Sleep outcomes (mean and 95% CI) for high school students (grades 9–12); (A) by race and year; (B) by FRL status and year.

The fitted linear mixed models using the ecological approach were used to examine whether differences in sleep outcome variables were modified by race or FRL status. Models for weekday and weekend sleep duration by level are found in Table 3, with weekday and weekend bedtime and wake time models by level found in Supplemental Table 2 (ST-2). Least square means for weekday and weekend sleep duration main effects are found in Table 4 (Race models) and Table 5 (FRL models), with means for weekday and weekend bedtime and wake time presented in Supplemental Table 3 (ST-3, bedtime) and Supplemental Table 4 (ST-4, wake time). Figures 2–4 present year-by-race and year-by-FRL interactions for all sleep variables by level, which are based on the ecological analytical approach unless otherwise noted (see Table 3 and ST-2 notes).

**Table 3.** *F*-Statistics and *p*-values for key predictors in linear mixed models for weekday and weekend sleep duration

	F*	*P*		*F* ^†^	*P*
Later elementary school (Grades 3–5)					
Weekday sleep duration			Weekday sleep duration		
Year	55.55	<0.0001	Year	43.76	<0.0001
Race	28.31	<0.0001	FRL	32.28	<0.0001
Race × year	2.41	0.0155	FRL × year	1.50	0.2304
Weekend sleep duration			Weekend sleep duration		
Year	0.32	0.7265	Year	0.01	0.9922
Race	2.00	0.0967	FRL	1.39	0.2457
Race × year	2.18	0.0290	FRL × year	1.73	0.1835
Middle school (Grades 6–8)					
Weekday sleep duration			Weekday sleep duration^‡^		
Year	121.55	<0.0001	Year	325.02	<0.0001
Race	6.12	0.0006	FRL	0.00	0.9812
Race × year	1.29	0.2607	FRL × year	2.72	0.0657
Weekend sleep duration			Weekend sleep duration		
Year	0.45	0.6468	Year	0.97	0.3969
Race	1.21	0.3201	FRL	3.36	0.0968
Race × year	2.09	0.0475	FRL × year	0.57	0.5756
High school (Grades 9–12)					
Weekday sleep duration			Weekday sleep duration		
Year	252.36	<0.0001	Year	202.31	<0.0001
Race	18.82	<0.0001	FRL	7.39	0.0418
Race × year	1.31	0.2656	FRL × year	1.15	0.3546
Weekend sleep duration			Weekend sleep duration		
Year	3.07	0.0911	Year	11.89	0.0023
Race	1.28	0.3124	FRL	4.94	0.0768
Race × year	3.22	0.0064	FRL × year	4.35	0.0436

Results for models with race are shown on left, those with FRL status on right. The ecological modeling approach was used to obtain results, unless otherwise noted.

*Numerator DF were as follows: Year = 2, Race = 4, Year × Race = 8; denominator DF ranged from 80 to 314.

^†^Numerator DF were as follows: Year = 2, FRL = 1, Year x FRL = 1; denominator DF ranged from 41 to 80.

^‡^Standard models did not converge, so for these conditions, so the unit modeling approach described in the methods section is reported.

**Table 4.** Means and 95% CI for weekday and weekend sleep duration main effects in race models (hours; year means averaged across race, race means averaged across years)

	Later elementary (Grades 3–5)		Middle school (Grades 6–8)		High school (Grades 9–12)	
	Weekday	Weekend	Weekday	Weekend	Weekday	Weekend
Year						
Pre-change	9.84 (9.80–9.89)	10.21 (10.16–10.27)	8.61 (8.56–8.67)	9.80 (9.72–9.87)	7.34 (7.27–7.41)	9.46 (9.36–9.57)
Post-change	9.62 (9.58–9.66)	10.19 (10.13–10.24)	9.09 (9.04–9.14)	9.75 (9.69–9.82)	8.12 (8.07–8.17)	9.40 (9.31–9.48)
Follow-up	9.59 (9.55–9.62)	10.18 (10.13–10.24)	9.05 (9.00–9.09)	9.78 (9.72–9.85)	7.96 (7.91–8.01)	9.37 (9.29–9.44)
Race						
White	9.81 (9.79–9.84)	10.22 (10.19–10.25)	8.98 (8.94–9.01)	9.81 (9.78–9.85)	7.86 (7.83–7.90)	9.43 (9.37–9.50)
Black	9.54 (9.48–9.59)	10.14 (10.05–10.22)	8.87 (8.81–8.92)	9.76 (9.68–9.84)	7.89 (7.79–7.99)	9.45 (9.32–9.58)
Hispanic	9.70 (9.66–9.75)	10.20 (10.14–10.26)	8.91 (8.86–8.96)	9.75 (9.68–9.83)	7.94 (7.88–8.01)	9.43 (9.33–9.53)
Asian	9.69 (9.63–9.74)	10.26 (10.18–10.34)	8.92 (8.87–8.97)	9.81 (9.72–9.90)	7.56 (7.49–7.63)	9.40 (9.27–9.54)
MR/AIAN/NHOPI	9.68 (9.62–9.74)	10.17 (10.10–10.24)	8.91 (8.86–8.97)	9.75 (9.64–9.87)	7.78 (7.72–7.85)	9.33 (9.22–9.43)

The ecological modeling approach was used to obtain results.

**Table 5.** Means and 95% CI for weekday and weekend sleep duration main effects in FRL status models (hours; year means averaged across FRL status, FRL status means averaged across years)

	Later elementary (Grades 3–5)		Middle school (Grades 6–8)		High school (Grades 9–12)	
	Weekday	Weekend	Weekday	Weekend	Weekday	Weekend
Year						
Pre-change	9.84 (9.80–9.88)	10.22 (10.18–10.27)	8.63 (8.58–8.68)	9.84 (9.77–9.91)	7.41 (7.34–7.49)	9.51 (9.38–9.63)
Post-change	9.65 (9.61–9.68)	10.22 (10.18–10.27)	9.14 (9.09–9.19)	9.82 (9.76–9.88)	8.17 (8.12–8.23)	9.49 (9.40–9.58)
Follow-up	9.62 (9.58–9.66)	10.23 (10.17–10.28)	9.06 (9.01–9.11)	9.79 (9.73–9.85)	8.01 (7.95–8.07)	9.38 (9.28–9.48)
FRL status						
FRL	9.63 (9.59–9.68)	10.24 (10.19–10.29)	8.95 (8.90–8.99)	9.84 (9.78–9.91)	7.89 (7.83–7.96)	9.50 (9.37–9.64)
Not FRL	9.77 (9.75–9.80)	10.21 (10.18–10.23)	8.94 (8.90–8.99)	9.79 (9.74–9.84)	7.84 (7.80–7.88)	9.41 (9.31–9.51)

The ecological modeling approach was used to obtain results.

For older ES students, no statistically significant interactions were observed for either race-by-year (Figure 2, A) or FRL-by-year (Figure 2, B) across all sleep outcomes on weekdays and weekends (Table 3 and ST-2). In both race and FRL models, a main effect was found for year (comparing findings by year, averaged across race, or FRL status), with significant changes similar to those previously reported in the descriptive findings for all weekday sleep outcome variables (i.e., earlier bedtime, earlier wake time, shorter sleep duration, all *p* < 0.0001), but no significant change in weekend sleep outcome variables across years (all *p* > 0.5). A main effect for race (comparing races averaged over years) was also found for all sleep variables (all *p* < 0.0001), except weekend sleep duration. Similarly, a main effect of FRL (comparing FRL status averaged over years) was found for weekday and weekend bedtimes, weekday sleep duration, and weekend wake times (all *p* < 0.0001). Means for all main effects can be found in Tables 4 and 5, ST-3, and ST-4.

For MS students, no statistically significant interactions were observed for either race-by-year (Figure 3, A) or FRL-by-year (Figure 3, B) across all sleep outcomes on weekdays and weekends (Table 3 and ST-2). A main effect for year, showing significant changes across the 3 years of the study, was found for all weekday sleep variables in both race and FRL models, as well as for weekend bedtime in the FRL model (all *p* < 0.0001). The direction of these changes was similar to previously reported descriptive results. A significant main effect for race was found for all sleep variables, except weekend sleep duration, while a significant main effect for FRL status was found only for weekend bedtime and wake time (all *p* < 0.0001). Main effect means are found in Tables 4 and 5, ST-3, and ST-4.

For HS students, no statistically significant interactions were observed for either race-by-year (Figure 4, A) or FRL-by-year (Figure 4, B) across all sleep outcomes on weekdays and weekends (Table 3 and ST-2). Interactions for race-by-year were borderline significant for weekend bedtime (*p* = 0.0019) and weekend sleep duration (*p* = 0.0064). Mean weekend bedtimes were later across all groups, with the smallest delay from pre-change to post-change in Black students (~11 min) and the largest delay in White students (~18 min). Weekend sleep duration changes were greatest for MR/AIAN/NHOPI students (14 min longer), with no change for Asian students. Significant main effects for year (all *p* ≤ 0.0001) were found for all weekday sleep variables and weekend bedtime in both the race and FRL status models, with borderline significant main effects for year found for weekend wake time in both models (*p* ≤ 0.004), and weekend sleep duration (*p* = 0.0023). The direction of changes in sleep outcomes were similar to those previously reported in the descriptive results. A significant main effect for race was found for weekday bedtime and weekday sleep duration (*p* < 0.0001), with no significant main effects for FRL status found. See Tables 4 and 5, ST-3, and ST-4 for all main effect means.

## Discussion

This is the first large scale study to concurrently examine the impact of changing school start times on sleep for primary and secondary school students in grades K-12, to provide follow-up data for 2 years post-change, to include both student and parent-proxy report, and to consider the impact on sleep by sociodemographic variables. Similar to previous studies [28, 33–39], results from CaSTLES confirm the significant benefit of later secondary school start times for MS and HS students’ sleep and daytime functioning, with benefits maintained for up to 2 years. Also similar to the one published study of US 3rd to 5th graders [25], CaSTLES found earlier bedtimes and wake times after changing to an earlier ES start time. As the earlier bedtimes were not proportional to earlier wake times, a small decrease in sleep duration was found. However, it is important to note that this 11-min difference is not considered clinically meaningful [22, 40], and the changes in the percent of ES students who obtained sufficient sleep duration or experienced daytime sleepiness was small. Furthermore, the reported post-change sleep duration remained consistent with previous studies of child sleep [18, 41, 42]. That said, it is critical for districts who are transitioning to earlier ES start times to provide education to families on the importance of sleep. As differences between racial and economic groups was found for weekday sleep variables, and with an increased recognition of sleep disparities that influence children’s sleep patterns [43, 44], steps should be taken to identify and ameliorate systemic factors that contribute to these differences. In addition, education programs should be developed in partnership with families to ensure that recommendations for adjusting bedtimes and increasing sleep opportunities are sensitive to sociocultural and environmental factors.

Sleep duration showed the greatest change for HS students, who obtained an extra 3.8 h per week, with MS students getting an extra 2.4 h of sleep per week. This resulted in a significant increase in the percent of students who obtained sufficient sleep after implementation of later start times (MS: pre 40.5%, post 61.0%, HS: pre 30.4%, post 62.7%). With ~15,000–20,000 MS and HS students per year in CaSTLES, this represents a large and significant number of students who are now obtaining sufficient sleep. The finding of this magnitude is in stark contrast to the more conservative goals and outcomes of Healthy People 2020 [45], which aimed to increase the percent of students obtaining sufficient sleep from 30.9% to 33.1% over 10 years. Unfortunately, the most recent national Youth Risk Behavior Survey reported that the percent of US adolescents obtaining sufficient sleep had actually *decreased* to 25.4% [46].

For MS and HS students, later weekday wake times were the most significant factor contributing to increased weekday sleep duration. One notable benefit of increased weekday sleep duration was the significant decrease in weekend oversleep, where a 2+ hour difference between weekday and weekend sleep duration is used as a marker of significant clinical sleep deprivation [1]. “Social jetlag” results from significantly shifting sleep–wake schedules on weekends, and has been associated with negative adolescent outcomes, including poorer mental health [16, 47, 48]. For HS students, the average weekend oversleep dropped from just over 2 h pre-change to 1.2 h post-change, suggesting that with sufficient weekday sleep, students are no longer clinically sleep deprived and needing to “catch up” on sleep on weekends. For adolescents with delayed sleep–wake phase disorder, another potential benefit of later start times, and thus later wake times, is better alignment with their natural circadian rhythm. Future reports should examine changes in sleep by chronotype (circadian preference).

Consistent with previous studies [27, 28, 33, 34, 36, 37, 39, 49, 50], weekday bedtimes for MS and HS students were not significantly or proportionately delayed with later start times, yet weekday wake times were significantly later. As weekend bedtimes were also minimally delayed, later weekday bedtimes were more likely a result of non-start time factors (e.g. developmental changes). Similar to ES students, racial differences in weekday bedtimes were found for both MS and HS students, suggesting again the importance of working with all families to identify and provide strategies, both structural and educational, for improving sleep health across populations of students.

For ES students, it was notable that parent-proxy and student-reported weekday wake times were nearly identical, highlighting the association between school start times and weekday sleep. However, compared to parent reports about their students, older ES students (grades 3–5) reported later weekday bedtimes (~15 min), later weekend bedtimes (~35–45 min), and later weekend wake times (~30 min). These findings highlight that parents may become less involved with bedtime and sleep routines as children get older [51–54]. In addition, the bedtime discrepancies may suggest that children do not fall asleep as early as parents believe.

While parents of MS and HS students also reported an increase in student sleep duration, parent-reported sleep duration was longer than student self-reported sleep duration. As parents and students reported similar student wake times, this discrepancy resulted from earlier parent-reported bedtimes for students (20–28 min difference). Similar to ES students, these findings support the fact that parental involvement with bedtime decreases with child age; however, parents often remain actively involved in waking adolescents on weekday mornings [55, 56].

Unlike previous studies, CaSTLES examined changes in sleep by socio-demographic variables (race and FRL status). Although there were significant differences between racial and economic groups for some sleep outcome variables, no significant interactions were found between sleep and socio-demographic variables over the 3 years of the study, suggesting that the change in start times did not impact racial and economic groups differently. For example, it is important to note that across racial groups, MS students reported an average increase of 24–33 min in weekday sleep duration, regardless of racial group, while high school students reported an average increase in sleep duration of 43–52 min. Although these data suggest benefits of later secondary school start times across student groups, it is important for future studies to continue examining potential sleep disparities.

Beyond sleep continuity (bedtime, wake time, duration), CaSTLES also examined the percent of students with clinically significant poor sleep quality and daytime sleepiness. For ES students, there was only a minimal decrease in sleep duration with earlier start times, with only a small percent of students reporting changes to sleep quality or daytime sleepiness. For MS students, no change in sleep quality was reported, however, there was a notable decrease in students reporting clinically significant daytime sleepiness (translating to ~1,100 students). For HS students, later school start times positively impacted both sleep quality and daytime sleepiness, with more than 1 in 10 students reporting improved sleep quality and 1 in 5 students reporting reduced daytime sleepiness post-change.

While this study has many strengths, several limitations should also be noted. First, all results are survey based, with no objective measures of sleep. However, having both student and parent reporters provides reasonable validity to the outcomes. Second, although data were collected across 3 years, only one survey per year (spring) was collected. It is possible that sleep patterns were different in the fall/winter, but using the same time frame window each year to collect survey data may mitigate the possible seasonal differences. Third, similar to previous studies, students in this study were not tracked across years, thus data were aggregated within a higher unit and it was not possible to tease out potential developmental factors that contributed to changes in outcomes. Fourth, this study did not include a control group. Although it is possible that sleep outcomes changed for reasons other than or in addition to different start times, the lack of change in sleep duration across the United States in the same time frame would suggest that later start times for secondary school students was the primary factor driving changes in sleep outcomes. Fifth, this paper focused only on sleep outcomes, thus no conclusions can be made at this time about the impact of changing school start times on academic, cognitive, or psychological outcomes. Finally, this study was conducted in suburban Denver, Colorado, and although the sample was somewhat diverse in terms of both race and socioeconomic status, the results may not be generalizable to either urban inner city school districts or rural school districts. That said, the examination of differences by sociodemographic variables suggests no significant negative sleep outcomes for changing to earlier start times across groups for ES students, and positive benefits of later start times across groups of MS and HS students.

## Conclusion

CaSTLES, a longitudinal study that includes a large, diverse sample, provides both complementary and novel findings to the literature on changing school start times. For secondary school students, recommended healthy school start times (at or after 8:30 am) result in increased sleep duration and decreased daytime sleepiness. In order to achieve these outcomes however, for many districts, “flipping” primary and secondary school start times is required to accommodate transportation schedules. CaSTLES findings support the option of moving primary school start times earlier, although future studies are needed to determine the optimal start time for younger students. Furthermore, when logistically and financially feasible, a uniform later school start time would be ideal for students and families.

Although recently passed legislation (CA Senate Bill 328) will implement healthy start times for all California secondary school students (starting fall 2022), many districts across the United States are still determining whether they should also change their bell schedules. This study provides critical evidence of how a single policy initiative, healthy school start times, is a significant and effective way to improve sleep duration and daytime sleepiness for large numbers of secondary school students. However, additional rigorous studies are needed to continue answering the many questions raised by changing school start times, most notably, how early is too early for primary school students to start their school day. Finally, as a sufficient sleep opportunity is critical for all students, education about the importance of healthy sleep patterns needs to be developed and disseminated to all families. In order to ensure that education programs are sensitive to factors that may contribute to sleep disparities (e.g. activities, parent or student work schedules, shared bedrooms), these programs should be developed in partnership with diverse students and parents.

## Supplementary Material

zsab048_suppl_Supplementary_Material
