# Supplementary material for: Changing school start times: impact on sleep in primary and secondary school students
Source: Sleep. 2021 Apr 15;44(7):zsab048. doi: 10.1093/sleep/zsab048 (PMC8271142; doi:10.1093/sleep/zsab048)
Supplement: zsab048_suppl_Supplementary_Material [file zsab048_suppl_supplementary_material.pdf]

Changing School Start Times: Impact on Sleep in Primary and Secondary School Students

Lisa J. Meltzer
National Jewish Health, 1400 Jackson Street, Denver, CO USA

Kyla L. Wahlstrom
University of Minnesota, 178 Pillsbury Drive SE, Minneapolis, MN USA

Amy E. Plog
Cherry Creek School District, 4700 S. Yosemite Street, Greenwood Village, CO USA

Matthew J. Strand
National Jewish Health, 1400 Jackson Street, Denver, CO USA

Address correspondence to:
Lisa J. Meltzer
National Jewish Health
1400 Jackson Street, G311
Denver, CO 80206
meltzerl@njhealth.org

Supplemental Table 1. Means (95% CIs) and differences for parent reported sleep outcome variables across years, based on descriptive analytical approaches.

	**Older Elementary School (Grades 3-5)**				
				Difference	
	Pre-Change	Post-Change	Follow-Up	Post – Pre	Follow-Up – Post
Bedtime[a]					
Weekday	20:55 (20:54 – 20:56)	20:46 (20:44 – 20:47)	20:45 (20:43 – 20:46)	-9 min	-1 min
Weekend	21:46 (21:44 – 21:47)	21:35 (21:33 – 21:37)	21:36 (21:34 – 21:39)	-11 min	+1 min
Wake Time[a]					
Weekday	7:06 (7:05 – 7:07)	6:40 (6:39 – 6:41)	6:41 (6:40 – 6:42)	-26 min	+1 min
Weekend	8:05 (8:03 – 8:07)	7:55 (7:53 – 7:58)	7:54 (7:51 – 7:56)	-10 min	-1 min
Duration[a]					
Weekday	10.18 hr (10.16 – 10.21)	9.90 hr (9.88 – 9.93)	9.94 hr (9.91 – 9.96)	-17 min	+2 min
Weekend	10.32 hr (10.29 – 10.35)	10.34 hr (10.31 – 10.38)	10.29 hr (10.25 – 10.33)	+1 min	-3 min
Weekend Oversleep[a,b]	8 min (6 – 10)	26 min (24 – 28)	21 min (19 – 23)	+18 min	-5 min
% Sufficient Sleep[c]	97.0%	94.5%	94.6%	-2.5%	+0.1%
% Poor Sleep Quality[d]	24.1%	23.3%	22.4%	-0.8%	0.9%
% Daytime Sleepiness[e]	32.3%	35.6%	34.7%	+3.3%	-0.9%

hr = hours, min = minutes

[a]Data are presented as mean (95% CI), with military time used for bedtimes and wake times

[b]Weekend oversleep is the difference between weekday and weekend sleep duration

[c]Sufficient sleep defined as an average of at least 9 hours

[d]Poor sleep quality defined as T $\geq$ 60 on PROMIS Pediatric Sleep Disturbance items

[e]Daytime sleepiness defined as T $\geq$ 60 on PROMIS Pediatric Sleep Related Impairment items

Supplemental Table 1 (continued). Estimated means (95% CIs) and differences for parent-reported sleep outcome variables across years, based on descriptive analytical approaches.

	Middle School (Grades 6-8)				
				Difference	
	Pre-Change	Post-Change	Follow-Up	Post – Pre	Follow-Up – Post
Bedtime[a]					
Weekday	21:26 (21:25 - 21:28)	21:32 (21:30 – 21:34)	21:34 (21:32 – 21:36)	+6 min	+2 min
Weekend	22:31 (22:29 – 22:32)	22:32 (22:30 – 22:34)	22:35 (22:33 – 22:37)	+1 min	+3 min
Wake Time[a]					
Weekday	6:31 (6:31 – 6:32)	7:07 (7:06 – 7:08)	7:06 (7:05 – 7:07)	+36 min	-1 min
Weekend	8:46 (8:43 – 8:48)	8:48 (8:46 – 8:51)	8:47 (8:45 – 8:50)	+2 min	-1 min
Duration[a]					
Weekday	9.08 hr (9.06 – 9.11)	9.58 hr (9.56 – 9.61)	9.53 hr (9.51 – 9.56)	+30 min	-3 min
Weekend	10.26 hr (10.22 – 10.29)	10.27 hr (10.23 – 10.31)	10.21 (10.17 – 10.25)	+1 min	-4 min
Weekend Oversleep[a,b]	1.17 hr (1.13 – 1.21)	0.69 hr (0.65 – 0.73)	0.67 hr (0.63 – 0.72)	-29 min	-1 min
% Sufficient Sleep[c]	64.4%	85.3%	83.6%	+20.9%	-1.7%

hr = hours, min = minutes

[a]Data are presented as mean (95% CI), with military time used for bedtimes and wake times

[b]Weekend oversleep is the difference between weekday and weekend sleep duration

[c]Sufficient sleep defined as an average of at least 9 hours

Supplemental Table 1 (continued). Estimated means (95% CIs) and differences for parent-reported sleep outcome variables across years, based on descriptive analytical approaches.

	**High School (Grades 9-12)**				
				Difference	
	Pre-Change	Post-Change	Follow-Up	Post – Pre	Follow-Up – Post
Bedtime[a]					
Weekday	22:03 (22:01 – 22:05)	22:15 (22:14 – 22:17)	22:18 (22:16 – 22:19)	+12 min	+3 min
Weekend	23:13 (23:11 – 23:15)	23:23 (23:21 – 23:25)	23:24 (23:22 – 23:26)	+10 min	+2 min
Wake Time[a]					
Weekday	5:53 (5:51 – 5:54)	6:49 (6:48 – 6:50)	6:50 (6:49 – 6:51)	+56 min	+1 min
Weekend	9:20 (9:17 – 9:23)	9:27 (9:24 – 9:30)	9:27 (9:24 – 9:30)	+7 min	no change
Duration[a]					
Weekday	7.83 hr (7.80 – 7.86)	8.56 hr (8.52 – 8.59)	8.54 hr (8.51 – 8.58)	+43 min	-1 min
Weekend	10.11 hr (10.07 – 10.16)	10.07 hr (10.02 – 10.11)	10.05 hr (10.01 – 10.09)	-3 min	-1 min
Weekend Oversleep[a,b]	2.28 hr (2.24 – 2.33)	1.51 hr (1.46 – 1.56)	1.51 hr (1.46 – 1.55)	-46 min	no change
% Sufficient Sleep[c]	49.2%	81.9%	80.4%	+32.7%	-1.5%

hr = hours, min = minutes

[a]Data are presented as mean (95% CI), with military time used for bedtimes and wake times

[b]Weekend oversleep is the difference between weekday and weekend sleep duration

[c]Sufficient sleep defined as an average of at least 9 hours

Supplemental Table 2. F-statistics and p-values for key predictors in linear mixed models for weekday and weekend bedtime and wake time. Results for models with race are shown on left, those with FRL status on right. The ecological modeling approach was used to obtain results, unless otherwise noted.

**Later Elementary School (Grades 3-5)**					
	F[a]	p		F[b]	p
Weekday Bedtime			Weekday Bedtime		
Year	19.56	<0.0001	Year	26.35	<0.0001
Race	54.05	<0.0001	FRL	50.51	<0.0001
Race x Year	2.09	0.0368	FRL x Year	0.30	0.7433
Weekday Wake Time			Weekday Wake Time		
Year	213.06	<0.0001	Year	232.38	<0.0001
Race	23.15	<0.0001	FRL	2.96	0.0931
Race x Year	2.49	0.0123	FRL x Year	1.05	0.3552
Weekend Bedtime			Weekend Bedtime		
Year	0.46	0.6315	Year	0.46	0.6315
Race	79.34	<0.0001	FRL	116.49	<0.0001
Race x Year	2.31	0.0203	FRL x Year	0.76	0.4726
Weekend Wake Time			Weekend Wake Time		
Year	0.39	0.6800	Year	0.66	0.5202
Race	55.13	<0.0001	FRL	109.77	<0.0001
Race x Year	1.55	0.1390	FRL x Year	0.34	0.7159

**Middle School (Grades 6-8)**					
	F[a]	p		F[b]	p
Weekday Bedtime			Weekday Bedtime		
Year	22.37	<0.0001	Year	15.90	<0.0001
Race	11.53	<0.0001	FRL	4.26	0.0660
Race x Year	1.31	0.2532	FRL x Year	2.49	0.1094
Weekday Wake Time			Weekday Wake Time		
Year	352.92	<0.0001	Year	289.8	<0.0001
Race	30.54	<0.0001	FRL	6.26	0.0313
Race x Year	3.28	0.0029	FRL x Year	0.02	0.9762

[a]Numerator DF were as follows: Year = 2, Race = 4, Year x Race = 8; denominator DF ranged from 80 to 314

[b]Numerator DF were as follows: Year = 2, FRL= 1, Year x FRL = 1; denominator DF ranged from 41 to 80

Supplemental Table 2 (continued). F-statistics and p-values for key predictors in linear mixed models for weekday and weekend bedtime and wake time. Results for models with race are shown on left, those with FRL status on right. The ecological modeling approach was used to obtain results, unless otherwise noted.

Middle School (Grades 6-8)					
	F[a]	p		F[b]	p
Weekend Bedtime			Weekend Bedtime[c]		
Year	4.01	0.0352	Year	16.38	<0.0001
Race	31.82	<0.0001	FRL	108.49	<0.0001
Race x Year	1.03	0.4232	FRL x Year	1.04	0.3552
Weekend Wake Time			Weekend Wake Time[c]		
Year	3.88	0.0388	Year	1.04	0.3517
Race	26.70	<0.0001	FRL	164.83	<0.0001
Race x Year	2.20	0.0368	FRL x Year	0.25	0.7777

High School (Grades 9-12)					
	F[a]	p		F[b]	p
Weekday Bedtime			Weekday Bedtime		
Year	234.65	<0.0001	Year	140.57	<0.0001
Race	51.69	<0.0001	FRL	12.86	0.0158
Race x Year	1.50	0.1887	FRL x Year	0.75	0.4967
Weekday Wake Time			Weekday Wake Time		
Year	995.98	<0.0001	Year	1349.72	<0.0001
Race	3.54	0.0243	FRL	0.47	0.5251
Race x Year	1.84	0.0970	FRL x Year	0.77	0.4865
Weekend Bedtime			Weekend Bedtime		
Year	24.98	0.0001	Year	25.36	0.0001
Race	3.22	0.0339	FRL	2.72	0.1600
Race x Year	3.86	0.0019	FRL x Year	1.19	0.3443
Weekend Wake Time			Weekend Wake Time		
Year	10.06	0.0040	Year	10.41	0.0036
Race	1.00	0.4318	FRL	11.35	0.0199
Race x Year	1.89	0.0882	FRL x Year	2.43	0.1381

[a]Numerator DF were as follows: Year = 2, Race = 4, Year x Race = 8; denominator DF ranged from 19 to 75

[b]Numerator DF were as follows: Year = 2, FRL= 1, Year x FRL = 1; denominator DF ranged from 10 to 19

[c]Standard models did not converge, so for these conditions, so the unit approach described in the methods section is reported

Supplemental Table 3. Means and 95% CI for weekday and weekend bedtime main effects (military time; Year means averaged across either Race or FRL Status, Race or FRL Status means averaged across Years). The ecological modeling approach was used to obtain results.

	**Later Elementary (Grades 3-5)**		**Middle School (Grades 6-8)**		**High School (Grades 9-12)**	
	Weekday	Weekend	Weekday	Weekend	Weekday	Weekend
Year						
Pre-Change	21:15 (21:13 – 21:18)	22:30 (22:25 – 22:35)	21:50 (21:47 – 21:53)	23:26 (23:19 – 23:34)	22:25 (22:23 - 22:27)	23:48 (23:41 - 23:54)
Post-Change	21:05 (21:03 – 21:08)	22:29 (22:24 – 22:33)	21:59 (21:57 – 22:01)	23:37 (23:31 - 23:44)	22:39 (22:37 - 22:40)	00:03 (23:58 - 00:08)
Follow-Up	21:07 (21:05 – 21:10)	22:31 (22:27 – 22:35)	22:01 (21:58 – 22:03)	23:35 (23:29 - 23:41)	22:47 (22:46 - 22:49)	00:05 (00:01 - 00:10)
Race						
White	20:59 (20:58 – 21:01)	22:06 (22:03 – 22:09)	21:52 (21:49 – 21:54)	23:19 (23:14 - 23:23)	22:33 (22:32 - 22:35)	23:56 (23:53 - 23:59)
Black	21:14 (21:10 – 21:17)	22:51 (22:45 – 22:57)	21:57 (21:54 – 22:00)	23:51 (23:45 - 23:56)	22:31 (22:28 - 22:35)	00:02 (23:56 - 00:08)
Hispanic	21:10 (21:07 – 21:13)	22:35 (22:31 – 22:40)	21:58 (21:55 – 22:00)	23:41 (23:34 - 23:48)	22:29 (22:27 - 22:31)	23:56 (23:49 - 00:04)
Asian	21:19 (21:16 – 21:21)	22:30 (22:24 – 22:35)	22:04 (22:00 – 22:07)	23:23 (23:14 - 23:31)	22:56 (22:53 - 22:59)	23:56 (23:46 - 00:06)
MR/AIAN/NHOPI	21:05 (21:02 – 21:09)	22:27 (22:20 – 22:34)	21:53 (21:50 – 21:57)	23:32 (23:24 - 23:41)	22:36 (22:33 - 22:40)	00:03 (23:55 - 00:11)

Supplemental Table 3 (continued). Means and 95% CI for weekday and weekend bedtime main effects (military time; Year means averaged across either Race or FRL Status, Race or FRL Status means averaged across Years). The ecological modeling approach was used to obtain results.

	**Later Elementary (Grades 3-5)**		**Middle School (Grades 6-8)**		**High School (Grades 9-12)**	
	Weekday	Weekend	Weekday	Weekend	Weekday	Weekend
Year						
Pre-Change	21:15<br>(21:12 – 21:18)	22:29<br>(22:25 – 22:34)	21:51<br>(21:48 – 21:54)	23:26<br>(23:18 – 23:34)	22:22<br>(22:19 - 22:25)	23:49<br>(23:42 - 23:56)
Post-Change	21:04<br>(21:01 – 21:06)	22:28<br>(22:24 – 22:32)	21:58<br>(21:55 – 22:00)	23:35<br>(23:27 - 23:43)	22:36<br>(22:34 - 22:38)	00:04<br>(23:58 - 00:09)
Follow-Up	21:05<br>(21:02 – 21:08)	22:30<br>(22:25 – 22:34)	22:00<br>(21:57 – 22:03)	23:36<br>(23:28 - 23:44)	22:44<br>(22:42 - 22:46)	00:07<br>(00:02 - 00:13)
FRL Status						
FRL	21:13<br>(21:10 – 21:16)	22:44<br>(22:39 – 22:49)	21:58<br>(21:55 – 22:01)	23:41<br>(23:32 - 23:49)	22:32<br>(22:29 - 22:35)	00:02<br>(23:54 - 00:10)
Not FRL	21:03<br>(21:01 – 21:05)	22:13<br>(22:10 – 22:17)	21:55<br>(21:52 – 21:57)	23:24<br>(23:16 - 23:32)	22:36<br>(22:34 - 22:38)	23:58<br>(23:53 - 00:02)

Supplemental Table 4. Means and 95% CI for weekday and weekend wake time main effects (military time; Year means averaged across either Race or FRL Status, Race or FRL Status means averaged across Years). The ecological modeling approach was used to obtain results.

	**Later Elementary (Grades 3-5)**		**Middle School (Grades 6-8)**		**High School (Grades 9-12)**	
	Weekday	Weekend	Weekday	Weekend	Weekday	Weekend
Year						
Pre-Change	7:06<br>(7:04 – 7:08)	8:44<br>(8:38 – 8:49)	6:27<br>(6:24 – 6:30)	9:14<br>(9:08 – 9:21)	5:46<br>(5:42 - 5:49)	9:14<br>(9:07 - 9:21)
Post-Change	6:43<br>(6:41 – 6:45)	8:41<br>(8:36 – 8:45)	7:05<br>(7:02 – 7:07)	9:23<br>(9:18 - 9:29)	6:46<br>(6:44 - 6:49)	9:26<br>(9:20 - 9:31)
Follow-Up	6:42<br>(6:41 – 6:44)	8:42<br>(8:37 – 8:47)	7:04<br>(7:02 – 7:07)	9:23<br>(9:18 - 9:28)	6:46<br>(6:43 - 6:48)	9:26<br>(9:21 - 9:31)
Race						
White	6:48<br>(6:47 – 6:49)	8:20<br>(8:16 – 8:23)	6:51<br>(6:49 – 6:52)	9:08<br>(9:04 - 9:12)	6:26<br>(6:23 - 6:28)	9:21<br>(9:18 - 9:25)
Black	6:46<br>(6:44 – 6:49)	8:59<br>(8:52 – 9:06)	6:50<br>(6:47 – 6:52)	9:38<br>(9:31 - 9:44)	6:25<br>(6:21 - 6:29)	9:27<br>(9:19 - 9:34)
Hispanic	6:52<br>(6:50 – 6:54)	8:48<br>(8:43 – 8:53)	6:52<br>(6:50 – 6:55)	9:26<br>(9:21 - 9:32)	6:26<br>(6:24 - 6:29)	9:22<br>(9:16 - 9:28)
Asian	7:00<br>(6:57 – 7:02)	8:46<br>(8:40 – 8:52)	6:59<br>(6:57 – 7:01)	9:11<br>(9:04 - 9:18)	6:29<br>(6:26 - 6:32)	9:19<br>(9:10 - 9:28)
MR/AIAN/NHOPI	6:46<br>(6:43 – 6:48)	8:38<br>(8:31 – 8:45)	6:48<br>(6:46 – 6:50)	9:19<br>(9:09 - 9:28)	6:24<br>(6:21 - 6:27)	9:20<br>(9:11 - 9:29)

Supplemental Table 4 (continued). Means and 95% CI for weekday and weekend wake time main effects (hours; Year means averaged across either Race or FRL Status, Race or FRL Status means averaged across Years). The ecological modeling approach was used to obtain results.

	**Later Elementary (Grades 3-5)**		**Middle School (Grades 6-8)**		**High School (Grades 9-12)**	
	Weekday	Weekend	Weekday	Weekend	Weekday	Weekend
Year						
Pre-Change	7:06<br>(7:04 – 7:08)	8:43<br>(8:38 – 8:48)	6:28<br>(6:25 – 6:30)	9:17<br>(9:10 – 9:23)	5:46<br>(5:43 - 5:50)	9:20<br>(9:14 - 9:25)
Post-Change	6:43<br>(6:41 – 6:44)	8:40<br>(8:36 – 8:45)	7:06<br>(7:03 – 7:08)	9:24<br>(9:17 - 9:31)	6:46<br>(6:43 - 6:49)	9:31<br>(9:27 - 9:35)
Follow-Up	6:43<br>(6:41 – 6:45)	8:43<br>(8:38 – 8:48)	7:04<br>(7:01 – 7:06)	9:23<br>(9:16 - 9:30)	6:45<br>(6:43 - 6:48)	9:29<br>(9:25 - 9:34)
FRL Status						
FRL	6:51<br>(6:49 – 6:54)	8:58<br>(8:52 – 9:04)	6:53<br>(6:51 – 6:56)	9:32<br>(9:25 - 9:38)	6:26<br>(6:22 - 6:29)	9:32<br>(9:24 - 9:40)
Not FRL	6:49<br>(6:48 – 6:51)	8:26<br>(8:22 – 8:30)	6:51<br>(6:49 – 6:53)	9:11<br>(9:05 - 9:17)	6:26<br>(6:23 - 6:30)	9:22<br>(9:19 - 9:24)
